# Analysis of transportation patterns through call detail records (CDRs)

**DOI:** 10.1371/journal.pone.0330246

**Published:** 2025-09-16

**Authors:** Sarah Alhumoud

**Affiliations:** College of Computer and Information Sciences, Imam Mohammad Ibn Saud Islamic University (IMSIU), Riyadh, Saudi Arabia; Novum Research and Innovation Group, LEBANON

## Abstract

Public transportation is essential for smart city development, especially in rapidly growing urban areas. In Riyadh, Saudi Arabia, the implementation of the metro system is expected to significantly impact the city’s transportation dynamics. This study uses Call Detail Records (CDRs) to analyze human mobility patterns and predict metro usage in 2024. This study also developed a methodology to categorize Traffic Analysis Zones (TAZs) and create metro zones, aiding in the visualization of the city’s population distribution and expected flow around the upcoming metro stations. Demographic data, including information on females and non-Saudis, was incorporated to predict metro usage more accurately. This approach identified areas with higher anticipated metro demand for the metro and potential feeder bus routes to support transportation efficiency. The insights gained from this analysis contribute to optimizing the metro system and addressing the needs of target populations, such as women and non-Saudi residents. This study demonstrates how mobile phone data can enhance transport planning in emerging urban environments.

## 1. Introduction

**Fig 1:**
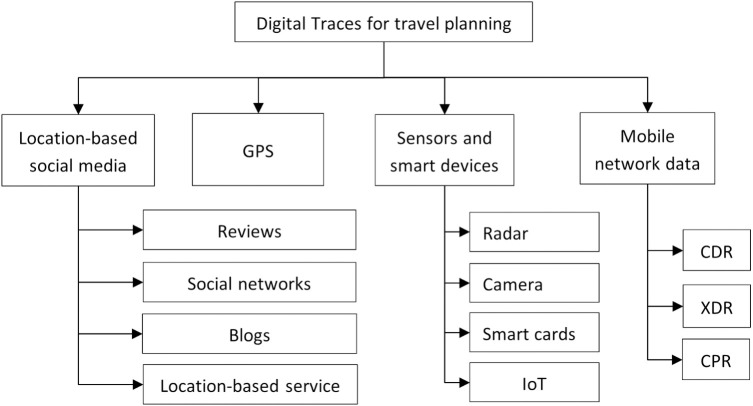
Digital traces for travel planning.

Rapid urbanization and population growth present significant challenges for cities worldwide, particularly in the domain of transportation planning. In Riyadh, the capital of Saudi Arabia, the establishment of a metro system has been identified as a pivotal solution to alleviate traffic congestion and promote sustainable urban mobility. As Riyadh is experiencing rapid economic, industrial, and tourism growth. According to Vision 2030, population growth and urbanization are projected to add 15–20 million people by 2030 [1]. The Riyadh Metro Project will help ease traffic congestion, including 84 metro stations and 6 metro lines [2]. However, designing an efficient metro system requires an in-depth understanding of mobility patterns and the diverse needs of the population. Manual surveys and traditional transportation analysis methods often fail to capture the dynamic and complex nature of urban mobility. This study addresses the critical gap in understanding how human mobility data can inform metro planning in a rapidly growing metropolis like Riyadh. Leveraging anonymized Call Detail Records (CDRs), it seeks to analyze mobility trends and identify high-demand areas that would benefit from metro accessibility. The primary objectives are to develop a robust framework for defining Traffic Analysis Zones (TAZs), assess potential metro utilization across demographic segments, and propose strategies for optimizing feeder bus networks to complement metro services. By addressing these objectives, the study contributes to Riyadh’s efforts to establish an integrated and sustainable urban transport system that meets the evolving needs of its diverse population. The latest research efforts in public transportation [3,4] are related to 1) Using Global Positioning Systems (GPS) data for tracking movements, 2) Using social media data, 3) Using sensors and smart devices data, 4) Using mobile network data such as Call Detail Records (CDRs) that capture call events, eXtended Detail Records (XDRs) that capture download/upload operations, or Control Plane Records (CPRs) that capture a network event. We composed big data resources for transportation, as shown in Fig 1.

Mobile phones are one of the most ubiquitous technologies of the modern era. The mobile penetration rate in Saudi Arabia is comparably high, reaching 130.5% in 2018, with 42.5 million subscriptions by the end of 2018 [5]. A CDR is a cellular network data that provides information about activities on a mobile communication network and aggregated human mobility. Utilizing travel data from mobile networks has been a valuable indicator in transportation development [6,7]; a CDR can capture the individual’s movement rate between sites and the potential transport links between those sites.

Unlike other sources, the CDR data collection process is generated by the user every time he makes or receives a phone call, and he does not control it; the user can’t disable tracking on the device. In addition, CDR data is huge and covers large geographical areas within cell stations. Thus, using CDR can solve specific issues related to the expected flow by estimating dynamic trajectories and the spatiotemporal distribution of people.

On the other hand, using CDR analysis for urban planning has some imitations and challenges [8,9]. A CDR provides the cell towers’ location and does not precisely determine the user’s location. In addition, the availability of data is restricted and needs a service provider’s permission. There is a lack of obtaining important contextual, demographic information for privacy reasons. Moreover, CDR only provides logged telecommunication operations, which means it does not track any user who do not make a call or receive a call. Lack of phone interaction may also lead to some estimation inaccuracies, as the user could stay idle in the middle of transit. Also, the user could hang. Furthermore, they lack the resolution to infer mode choices and have their own noise and biases that must be accounted for.

This study aims to leverage CDRs to derive insights into metro usage across 84 stations in Riyadh. The study’s objectives are; 1) To determine whether the station sites are ideal. 2) To determine which metro stations most need feeder buses. 3) To investigate the expected metro flow. The study focuses on women and non-Saudi residents as the most targeted beneficiaries of the metro. As of 2020, only 2% of women had been issued a driving license. One of the major factors that prevented them from obtaining a license was the cost of car ownership [10].

## 2. Related works

Mobile data for travel demand estimation has been explored in several studies. Passively collected data from mobile devices can reflect individuals’ travel patterns and predict travel demand.

Alhasoun et al. [6] proposed a dynamic Bayesian network approach to couple the location sequences of the individuals under consideration with those of their “similar strangers”. The paper investigated the temporal and spatial similarity of users’ mobility patterns. It developed a dynamic Bayesian network incorporating other users’ information with similar mobility characteristics. This was done to improve the accuracy of the next location prediction for a given user. The CDR dataset provided by Saudi Telecom Company (STC) in Saudi Arabia for the city of Riyadh, gathered from around 1800 towers, includes the user ID of the caller and receiver, the type of communication (i.e., SMS, MMS, call, data), the cell tower ID facilitating the service, the duration, and a timestamp of the phone activity). Compared to Lu et al. [11] paper, the proposed model performed better than the Markov chain-based models when human movement becomes more unpredictable, and the interaction between similar strangers is more advantageous.

Jiang et al. [12] used Singapore as an example to show how big data analytics may translate ubiquitous mobile phone data into a planner interpretable human movement pattern. This is done by estimating trip plans at the individual level. The authors applied trip-based and activity-based approaches to quantify the spatial distribution of travel patterns by residents in different city areas. These approaches helped to predict an individual’s frequently visited sites and, ultimately, their stay locations accurately for up to 90% of the population. According to the study, individual mobility patterns, which are significant for urban planning, can be anticipated using big data. These mobility estimates are more robust, less expensive to predict, and cover a larger geographical area than typical approaches.

Hankaew et al. [13] utilized mobile phone network data to infer migration trips and travel to predict human mobility. Long-distance journeys (such as migration) are generally less frequent than small trips like shopping trips and are poorly represented in most regional and national travel demand models. Since long-distance trips account for a significant portion of total kilometers traveled, particularly on high-capacity lines, infrastructure for long-distance excursions is required, making it critical to account for travel demand models. Insightful aspects of inferred migration trips are considered, such as intra/inter-district migration flows, migration distance distribution, and Origin-Destination (O-D) movements. Log-linear, classical gravity, and recently developed radiation models were examined using CDR to model migration trip distribution. Various approaches were used to define each model’s parameters. The authors concluded that radiation models perform less effectively in such a setting than log-linear and gravity models.

Sakamanee et al. [14] investigated route selection and mobility patterns between home and work. The study added to the transportation modeling literature by proposing a novel method for gathering crowdsourced route choice behavior data. The study used CDR data to infer individual route selection for commuting trips for 110,213 users in Portugal. The primary methods rely on route waypoint interpolation, the shortest distance between a route choice and a mobile usage location, and Voronoi cells, which divide a route choice into coverage zones. The four-step model consists of trip generation, trip distribution, mode choice, and route assignment to understand mobility patterns with CDR. In the trip generation step, CDR is employed to infer trip volume and geographical distribution. This is for calculating commuting trip generation rates, calibrating a hybrid trip generation model, and assessing zonal travel demand. CDR is used in O-D matrix building, evaluating, and modeling in the trip distribution step. CDR data is utilized to infer commuting mode choices based on the distance between cell towers, route choices, travel time, and weak labeling of visited cell towers. At the route assignment stage, CDR is utilized to infer individual route selection for commuting trips. The study added to the literature by suggesting using CDR data at street level, which has rarely been explored.

Tanberk and Demir [15] developed a novel approach based on the usage and time slot information provided by CDR data. The aim was to utilize data faster while maintaining manageable and practical technology. By analyzing the CDRs, telecom operators were able to predict how mobile users are going to behave in the next slot. The study suggested a novel approach using the Long-Short Term Memory Network (LSTM) and Extreme Gradient Boosting (XGBoost) algorithms. While the LSTM model forecasts traffic data in multivariant time series, the XGBoost model outperformed it.

Jiang et al. [16] modeled urban mobility with TimeGeo, a framework that separates individual characteristics and key mechanisms required to construct entire urban mobility profiles from imperfect data available in telecommunication operations. Using mobile data, TimeGeo creates an individual mobility profile between home and work. This includes stay duration, number of visits, daily mobility network, and types of activities that individuals engage in depending on the time of their visits. By labeling visited location types for individual users as home, work, or other, representative traffic O-D matrices were created for an average day and by the time of day. The study demonstrated how these characteristics of population distribution can be derived from large amounts of data instead of employing social-demographic information.

Salgado et al. [17] created an urban science framework to characterize phone users’ exposure to various street environment types, specifically for diverse ethnic groups (Whites, Blacks, Hispanics, and Asians) using network science, Geographical Information Systems (GIS), daily individual trajectories, and street images. Street network visualization helped characterize a city’s underlying mobility. Incorporating human mobility data into street networks helped assess congestion during peak hour traffic and road demand. They combined the advantages of both perspectives and helped understand the significance of streets in terms of the opportunities they provide and the demand they receive. Street imaging was used to detect land use, street context, neighborhood income, perceived safety, and car accident risk. Artificial Intelligence (AI) [11] helped classify images effectively and massively. A street network analysis revealed extensive routes linking different city regions. The authors suggested that this type of analysis can improve urban science methodologies based on street appearance by indicating where to focus (via demand) and reducing working costs (via AI), allowing for more accessible cities, and making identifying inequalities between different demographic groups easier.

Cuttone et al. [18] examined human mobility using CDR, GPS, WiFi, travel surveys, and various methodologies for predicting models, such as Markov chains, Naive Bayes, artificial neural networks, and time series analysis. Studies indicated varying results for the predictive capacity of these models, with accuracy ranging from over 90% to less than 40%. The study attempted to investigate the factors that impact the predictability of models, such as next-place prediction, spatial resolution prediction, time resolution, the dataset’s demographics, and the exploration of new places.

Table 1 summarizes related work in terms of the study context, the targeted location of the study, the dataset used, and the method. Based on previous studies [6,7], CDRs can be an excellent resource for creating urban travel models. On the other hand, CDRs do not provide the details and contextual information that surveys do. This includes demographic information about the travelers and the purpose and mode of transportation. In addition, CDR data is not guaranteed to give complete trip information. This is because cell phone data only tracks where a phone call began and ended. Although data like surveys, GIS, or GPS are not provided in our study, creating O-D metrics will enable us to filter out noise from CDR to extract meaningful data. Moreover, an algorithm could be used to determine the locations specific users regularly visit for further insight and analysis. These locations are categorized as home, work, or other based on visits times.

**Table 1:** Related works.

Article	Context	Location	Data	Method
Alhasoun et al. (2017)	Reduce the effects of the sparsity in the data and improves the accuracy of predicting the next location	Riyadh, Saudi Arabia.	STC CDR dataset from 1800 towers (dataset size N/A).	Dynamic Bayesian network
Jiang et al. (2017)	Use CDR data to extract individual mobility networks to estimate travel plans at an individual level.	Singapore, Singapore.	Household interview travel survey from 34,000 individuals and their 1-day travel information (trip arrival and departure time, trip purpose, location of trip origins and destinations) + CDR data collected for 2 weeks between March and April 2011 with 3.17 million users.	A trip-based approach to generate algorithms to identify individual stay locations, and an Activity-based approach to estimate stay locations for individuals.
Hankaew et al. (2019)	Predict migration-based flows, migration-inferred trips and movements at a national level.	Portugal	CDR data from one carrier in Portugal contains 1,891,928 mobile phone users (around 18% of the population) for 14 months (from 2 April 2006 until 30 June 2007).	Gravity model, Log-linear model, and Radiation model.
Sakamanee et al. (2020)	Determine the route choice between home and workplace based on CDR data.	Portugal	CDR data contains 1.8 million user records from April 2006 to March 2007. Only 110,213 users connected to cellular networks at least five times each month were chosen. The dataset includes 6,511 cell towers.	Interpolation-Based Methods, Shortest Distance-Based Methods, and Voronoi Cell-Based Methods.
Tanberk and Demir (2022)	Propose a new data structure out of the CDR data to increase the efficiency of models’ prediction.	Milan, Italy	CDR Data includes 62 days between November 2013 to January 2014.	LSTM and XGBoost.
Jiang, et al. (2016)	Generate mobility profiles of individuals based on home and work location including stay duration, number of visits, and daily mobility network.	Greater Boston, USA	CDR data contains 1.92 million users in the Greater Boston area over a 6-week period in 2010 + a set of self-collected mobile phone traces across 14 months in 2013 and 2014.	TimeGeo modeling framework, Markov model, and Kolmogorov–Smirnov (KS) test statistic.
Salgado et al. (2021)	Investigate each racial/ethnic group’s (White, Black, Hispanic and Asian) street demand based on street networks.	Boston, USA	CDRs from Jiang et al. (2016) + City demographic information including the number of self-reported persons in the racial/ethnic groups of White, Black, Hispanic, and Asian + Two sets of street imagery from Google Street View (23, 927, and 5998 images respectively).	AI labeling methodology and CDR-based TimeGeo models are used.
Cuttone (2018)	Understand the factors that improve the accuracy of predictive models for human mobility.	Copenhagen, Denmark	GPS location, Bluetooth, SMS, phone contacts, WiFi, Facebook friendships + CDR data for 454 users (collected between 2012–2013).	Toploc technique, the Markov chain model, and the logistic regression approach.

## 3. Methodology

In this research, we investigate the expected flow of the metro stations and if the proposed metro stations’ location is optimal and reachable. We present the dataset in section 3.1 to explain the various types of data under study, which were critical factors in analyzing the metro zone system. In addition, in section 3.2, we analyze CDR data to get an insight into the aggregate movement of people. Moreover, we propose an algorithm in section 3.3 to define the metro zones. This algorithm determines which metro station is closest to each geography unit and most reachable via the road network.

### 3.1. Dataset

**Fig 2:**
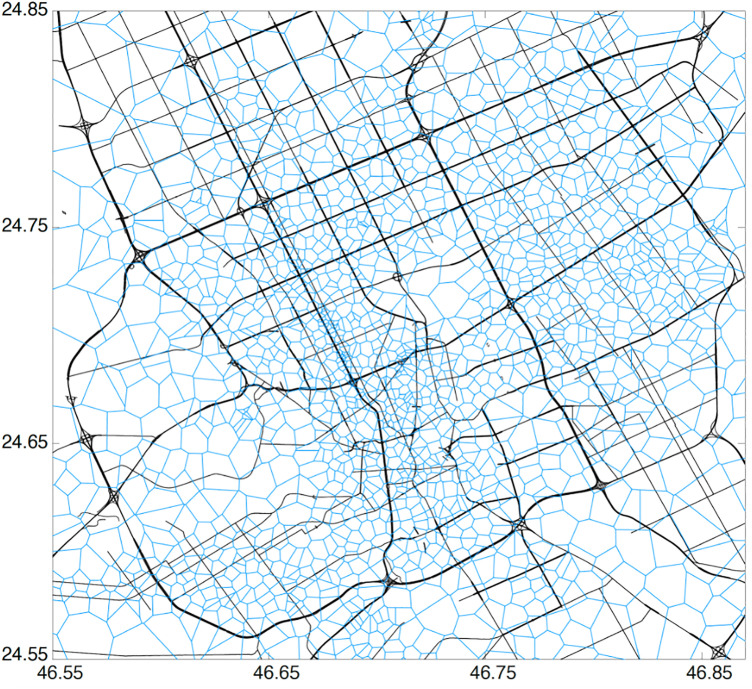
Voronoi cells corresponding to each cell tower coverage.

The dataset utilized in this study comprises one month of Call Detail Records (CDRs) from Riyadh, Saudi Arabia, encompassing approximately 1,800 cell towers. The data, provided by STC, a telecom operator, represents phone activity during December 2012. Over this period, approximately 300,000 users generated about 109 million records. The spatial granularity of the cell towers varies across Riyadh, as depicted by Alhasoun et al. [1] in Fig 2, with smaller cell sizes near the city center and larger cells in peripheral areas.

#### 3.1.1. CDR.

CDR data represents a digital record containing information about a telephone call or communication session. A CDR typically includes information such as the caller’s and recipient’s phone numbers, the date and time of the call, the duration of the call, and any additional services or features used during the call (e.g., call forwarding, call waiting). In addition, CDRs may contain information about the type of call (voice, video, data), the caller’s or recipient’s location, and details about any supplementary services utilized during the call.

#### 3.1.2. TAZ.

Traffic Analysis Zones (TAZs) are geographical areas defined and used for transportation planning and traffic analysis purposes. TAZs are created by dividing a large region or area into smaller sub-areas based on population density, land use patterns, transportation infrastructure, and socio-economic characteristics. TAZ data are provided by the royal commission of Riyadh city (RCRC) [2]. TAZs are defined to facilitate transportation planning and analysis by providing a more granular and manageable unit for studying travel patterns and forecasting transportation demand.

TAZs are often used in transportation models and simulations to estimate and analyze traffic flows, travel behavior, and travel demand within specific areas. As part of this study, Riyadh city is defined as 1492 TAZ.

#### 3.1.3. Metro stations.

In this study, we consider 84 metro stations. Based on the spatial information of each TAZ, we associated each TAZ with the closest metro station. This made it easier to predict metro usage.

#### 3.1.4. Demographic data.

The demographic data is available in TAZ. Each TAZ includes data on the total population of males, females, and non-Saudi residents. Females and non-Saudis are expected to utilize the metro more based on the sociocultural implications of the region. They were integrated with CDR data based on Origin-Destination (O-D) movements between TAZ Voronoi cells. As explained in section 3.3. Hence, areas with higher concentrations of those populations expect more metro usage.

#### 3.1.5. Road network.

The road network consists of several thousand lines. The lines are represented by many points defined by latitude and longitude. Each of these points represents a node. The road network was used to creat metro zones as will be explained in section 3.4.

### 3.2. CDR analysis

Approximately 100,000 distinct sources with only a single trip during the month were excluded from the analysis. Next, we calculated which TAZ and metro zone each cell phone tower belonged to based on the tower’s latitude and longitude. Then we created the files that put the CDRs in terms of TAZs and metro zones.

The O-D matrix was constructed by extracting all source destinations and their flows for users who frequently make trips. Then, number of call records were analysed each time to determine when people travel most frequently. This could indicate when the metro will be busy. CDR data is also incorporated into the visualizations. Lines show the overall highest flows in this view both out of all the data and at specific times. This enabled clear identification of the times and locations where metro zones were most used.

Furthermore, this was incorporated with the demographic data available in TAZ, allowing to make more predictions about the expected metro usage. For example, if a source and destination had very high flow but mainly Saudi males, it would indicate some metro usage. However, not as much as if the areas were females and non-Saudi residents.

### 3.3. Metro zones

Metro zones are clusters of nearby TAZs. Each metro zone has a unique metro stop associated with it. Metro zones were created to correlate demographic data to metro stations and obtain more aggregated data to notice trends affecting metro usage.

To create metro zones, first, we find the most appropriate node on the road network to represent the TAZ; this could be done by finding the TAZ centroid. Next, we determined which road node it was closest to (by Euclidean distance). The metro stops were also defined as polygons, so we did the same process with those to determine which road node best represented the metro station. We then ran Dijkstra’s algorithm from each TAZ starting point. Then, for each TAZ, we found the metro station road node with the minimum shortest path. We classified TAZ as part of the metro station’s metro zone. Note that the shortest path algorithm was run on all road nodes. However, the only final distances were those nodes associated with a metro station.

**Fig 3:**
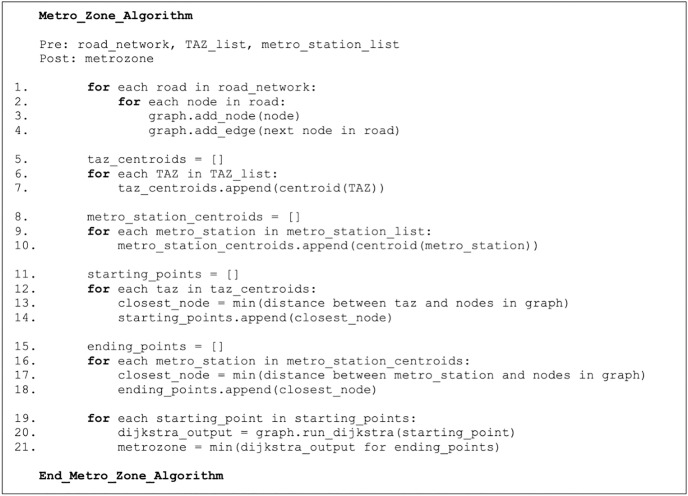
The metro zone algorithm pseudo-code.

The summary of the steps of the algorithm in Fig 3 is as follows:

1. Find the centroid of each TAZ.
2. Create a graph of Riyadh’s road network.
3. Determine which node in the graph is closest to each TAZ centroid (start points).
4. Determine which node in the graph is closest to each metro station’s centroid (end points).
5. Run Dijkstra’s shortest path algorithm from each starting point.
6. Find the shortest path between each starting point and the corresponding end point and assign the corresponding TAZ to the corresponding metro zone

The algorithm has some limitations: each TAZ location is characterized by its centroid, and the road network used to build the graph is not perfectly distributed.

### 3.4. Ethical statement

This research entails mathematical computation involving Call Detail Records (CDRs) to look at human movement in Riyadh. To ensure individual privacy, data used in this study was anonymized. For all the researched information, no personally identifiable information was utilized in the process. The permission of the usage of CDRs for this study was obtained from the proper telecommunications service provider (Saudi Telecom Company) and was an adherence to data protection laws of the country. Further, since the present research does not involve regular human subjects or that would be interacting with them directly, the research is exempted from approval by the institutional review board (IRB). However, all the research processes adhered to the national and international ethics with the use of the anonymized personal information.

## 4. Results and discussion

This section offers visualized metro zones and demographic information available in the TAZ to identify areas with large female and and non-Saudi residents population since they will likely use the metro frequently.

### 4.1. Visualization of metro zones

**Fig 4:**
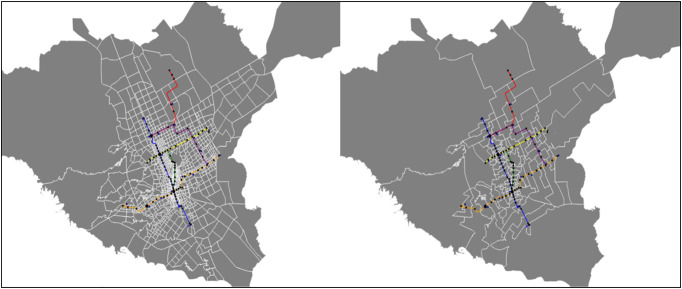
The left image shows Riyadh Split by TAZ with the proposed metro system. The right image shows Riyadh split by the metro zones created from the proposed algorithm.

Fig 4 compares the original TAZs with the metro zones created by the algorithm. After visualizing the results, we found that despite these limitations, the algorithm was sufficient to categorize the TAZs for our purposes.

### 4.2. Visualization of demography

Using a dashboard, we visualized the map with different options, including demographic data based on TAZs and metro zones presented as percentages and numbers.

The light blue area represents fewer Saudi females and and non-Saudi residents, while the dark blue area represents more Saudi females and and non-Saudi residents. This could be determined by the number of females and and non-Saudi residents in the area or by percentage.

The option to view both percentages and numbers is provided because, even in a zone with a low percentage of females and and non-Saudi residents but a very high total population, there could be more of the target population than in zones with a high rate but low total population. The option to view a chart of only non-Saudi or female populations was also available. Additionally, we can view the population by TAZ or metro zone. The wide range of options provides valuable insights into population spread.

**Fig 5:**
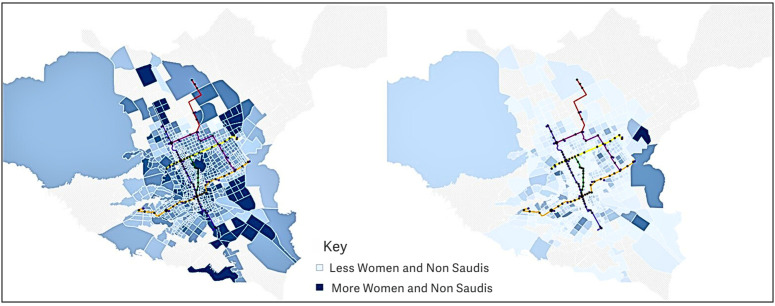
Demography by TAZ. The image on the left shows the data by percentage, darker regions correspond to a higher percentage of non-Saudis and females in that TAZ. The image on the right shows data by number, darker regions correspond to higher populations of females and non-Saudis.

**Fig 6:**
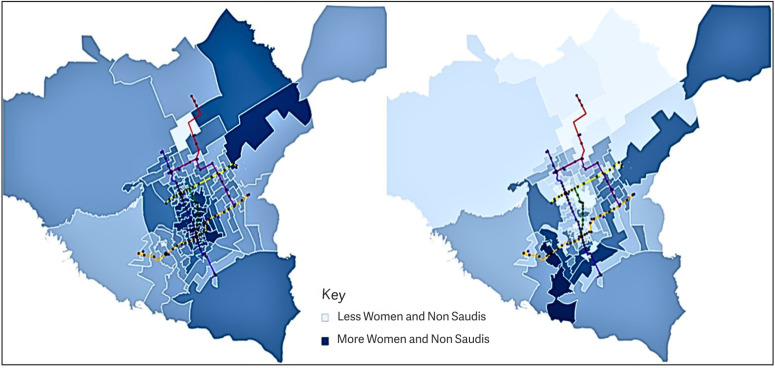
Demography by metro zone. The image on the left shows the data by percentage, darker regions correspond to higher percentages of non-Saudis and females in that metro zone. The image on the right shows data by number, darker regions correspond to higher populations of females and non-Saudis.

Metro stations more likely to be sources of trips can be specified by identifying large concentrations of females and non-Saudi residents. In these views, there are many females and non-Saudis along the metro lines, so it will be helpful to utilize them. According to the chart views, many areas towards the ends of the metro lines had higher female and non-Saudi populations, specifically at the eastern end of the yellow and orange lines and at the southern end of the blue line. Additionally, iconic stations are presented as large black circles. Those stations along the orange line have significant female and non-Saudi populations. Figs 5 and 6 show the choropleth view of demography by TAZ and metro zone, respectively. The large populations of these demographic groups, especially towards the ends of the lines, suggest that these will be popular origins for metro trips.

### 4.3. Visualization of CDR-based most popular trips

**Fig 7:**
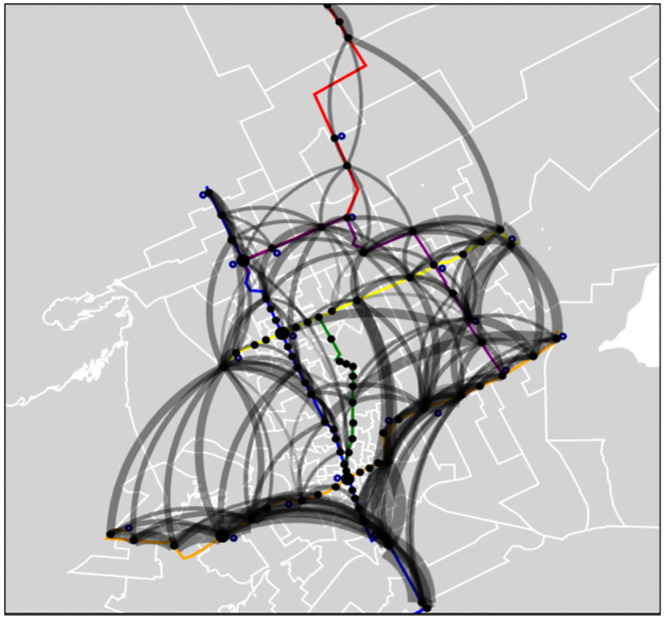
Most popular trips taken between metro zones (according to CDRs).

While the demographic data tell us where people live, the CDR data tell us where those people were going, which provides more information about the expected flow. Fig 7 shows the most prevalent CDR flows from all hours over 30 days; metro zones categorize CDR sources and destinations. Sources and destinations have been mapped to the metro station corresponding to their metro zone as shown in the visualization. The thickness of the lines indicates the popularity of the trip based on the number of people who took the trip. Based on the CDR, the minimum number of trips was 1, the average was 221, and the maximum number was over 15,000 trips. However, the visualization displays only trips taken more than 750 times over 30 days, as these were the top 5% most popular trips. In addition, all trips from that destination to that source had large flows, indicating that these are significant trips and not just mid-trip calls.

Categorized areas with the most popular trips can be observed. Trips originating or ending in the southern half of the blue line and along the orange line are common. The westmost stop on the yellow line and the eastern stops on the yellow line appear popular. Metro zones are large geographical areas, especially those on the city’s outskirts; we cannot assume that trips beginning in one metro zone are near that metro station. We can view the trips with the sources and destinations as the nearest TAZ to get a more fine-grained look at the CDR data.

**Fig 8:**
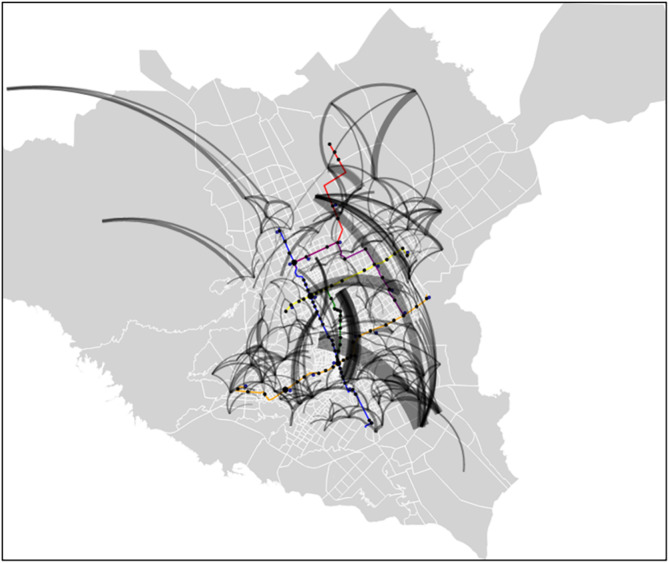
Most popular trips by TAZ (according to CDRs).

Fig 8 shows the most popular trips made by the closest TAZ during the 30 days. The thicker lines represent a more popular trip. There is, however, a difference in scale between Figs 7 and 8. Only trips with over 150 flows are displayed in Fig 8. This visualization gives us additional insight into where people travel throughout the city. Although some trips are outside the metro system’s range, especially on the city’s western side, most are in the city’s central area with a metro station near the source or destination. One of the city’s most famous source/destination lines is in the southern part, as it is depicted in Fig 7. The corresponding sources/destinations for trips are mostly near metro stations. This area could be an ideal candidate for a feeder bus to the blue line. These visualizations and the CDR data show that the blue and orange lines are likely to be the most popular.

According to our analysis of the timing of the trips, the most popular travel hours were between 4 and 9 pm. The most popular hour was between 6 pm and 7 pm, with just under 7.5% of trips taken. The hours from 1 am to 6 am had the least number of calls. Each hour contains less than 1% of the total data since traveling in the middle of the night is rare. Considering this data, the metro system is likely to be the most crowded in the evening.

### 4.4. Results summary

The study presented several significant findings. A robust methodology was developed to categorize Traffic Analysis Zones (TAZs) based on mobility patterns derived from Call Detail Records (CDRs), which enabled the identification of metro zones and prioritized areas with high potential for metro usage. High anticipated metro usage was observed among females and non-Saudis, highlighting these groups as primary users of public transport. Specific metro lines with the greatest potential demand were identified, offering guidance for planning priorities. The study also mapped regions with high expected ridership, emphasizing areas that would benefit from feeder bus networks to enhance metro access. Additionally, optimal travel times for metro usage were determined, providing actionable insights for scheduling and operational planning. By integrating demographic data into the mobility analysis, the study offered a nuanced understanding of the needs of diverse user groups. Lastly, the feasibility of using CDRs as a reliable data source for transportation planning was validated, reinforcing the utility of this innovative approach in addressing Riyadh’s urban mobility challenges.

## 5. Conclusions and future directions

This study utilized Call Detail Records (CDRs) to analyze human mobility patterns in Riyadh, aiming to predict metro usage and optimize transportation planning. The research developed a novel methodology for categorizing Traffic Analysis Zones (TAZs) and defining metro zones, which integrated demographic data to identify high-demand areas and potential feeder bus routes. Key findings include identifying metro lines with high anticipated utilization, particularly among females and non-Saudis, and determining optimal travel times for metro usage.

The insights gained highlight the potential of mobile phone data as a powerful tool for transport planning in rapidly urbanizing regions. However, limitations such as data resolution, and the inability to capture all mobility patterns underline the need for supplementary data sources, like geospatial and sentiment data.

Future research could focus on incorporating real-time data from additional sources, such as social media and GPS, to enhance the precision of mobility predictions. Investigating temporal variations and integrating points of interest into the analysis could further contextualize travel behaviors. Moreover, exploring the impact of road traffic conditions and metro station accessibility on ridership could refine feeder bus network planning. These advancements will support Riyadh’s vision for a smart and sustainable transportation system.
